# Ensemble Deep-Learning-Based Prognostic and Prediction for Recurrence of Sporadic Odontogenic Keratocysts on Hematoxylin and Eosin Stained Pathological Images of Incisional Biopsies

**DOI:** 10.3390/jpm12081220

**Published:** 2022-07-27

**Authors:** Roopa S. Rao, Divya Biligere Shivanna, Surendra Lakshminarayana, Kirti Shankar Mahadevpur, Yaser Ali Alhazmi, Mohammed Mousa H. Bakri, Hazar S. Alharbi, Khalid J. Alzahrani, Khalaf F. Alsharif, Hamsa Jameel Banjer, Mrim M. Alnfiai, Rodolfo Reda, Shankargouda Patil, Luca Testarelli

**Affiliations:** 1 Department of Oral Pathology and Microbiology, Faculty of Dental Sciences, Ramaiah University of Applied Sciences, Bengaluru 560054, India; drroopasrao1971@gmail.com (R.S.R.); drsuri29@gmail.com (S.L.); 2 Department of Computer Science and Engineering, Faculty of Engineering and Technology, Ramaiah University of Applied Sciences, Bengaluru 560054, India; divyabies@gmail.com (D.B.S.); mkirtishankar@gmail.com (K.S.M.); 3 Division of Oral and Maxillofacial Surgery, Department of Oral and Maxillofacial Surgery and Diagnostic Sciences, College of Dentistry, Jazan University, Jazan 45412, Saudi Arabia; dr.y.alhazmi@gmail.com (Y.A.A.); mmb644@nyu.edu (M.M.H.B.); 4 Department of Basic Dental Science, College of Dentistry, Princess Nourah Bint Abdulrahman University, Riyadh 11564, Saudi Arabia; hasalharbi@pnu.edu.sa; 5 Department of Clinical Laboratories Sciences, College of Applied Medical Sciences, Taif University, 11099, Taif 21944, Saudi Arabia; ak.jamaan@tu.edu.sa (K.J.A.); alsharif@tu.edu.sa (K.F.A.); h.banjer@tu.edu.sa (H.J.B.); 6 Department of Information Technology, College of Computers and Information Technology, Taif University, 11099, Taif 21944, Saudi Arabia; m.alnofiee@tu.edu.sa; 7 Department of Oral and Maxillo Facial Sciences, Sapienza University of Rome, 00161 Rome, Italy; luca.testarelli@uniroma1.it; 8 Department of Maxillofacial Surgery and Diagnostic Sciences, Division of Oral Pathology, College of Dentistry, Jazan University, Jazan 45412, Saudi Arabia; 9 Centre of Molecular Medicine and Diagnostics (COMManD), Saveetha Dental College & Hospitals, Saveetha Institute of Medical and Technical Sciences, Saveetha University, Chennai 600077, India

**Keywords:** deep learning, machine learning, microscopy, odontogenic keratocysts, oral, pathology, prognosis, sporadic form

## Abstract

(1) Background: Odontogenic keratocysts (OKCs) are enigmatic developmental cysts that deserve special attention due to their heterogeneous appearance in histopathological characteristics and high recurrence rate. Despite several nomenclatures for classification, clinicians still confront challenges in its diagnosis and predicting its recurrence. This paper proposes an ensemble deep-learning-based prognostic and prediction algorithm, for the recurrence of sporadic odontogenic keratocysts, on hematoxylin and eosin stained pathological images of incisional biopsies before treatment. (2) Materials and Methods: In this study, we applied a deep-learning algorithm to an ensemble approach integrated with DenseNet-121, Inception-V3, and Inception-Resnet-V3 classifiers. Around 1660 hematoxylin and eosin stained pathologically annotated digital images of OKC-diagnosed (60) patients were supplied to train and predict recurrent OKCs. (3) Results: The presence of SEH (*p* = 0.004), an incomplete epithelial lining, (*p* = 0.023), and a corrugated surface (*p* = 0.049) were the most significant histological parameters distinguishing recurrent and non-recurrent OKCs. Amongst the classifiers, DenseNet-121 showed 93% accuracy in predicting recurrent OKCs. Furthermore, integrating and training the traditional ensemble model showed an accuracy of 95% and an AUC of 0.9872, with an execution time of 192.9 s. In comparison, our proposed model showed 97% accuracy with an execution time of 154.6 s. (4) Conclusions: Considering the outcome of our novel ensemble model, based on accuracy and execution time, the presented design could be embedded into a computer-aided design system for automation of risk stratification of odontogenic keratocysts.

## 1. Introduction

Odontogenic cysts are one of the most prevalent lesions that make up a significant portion of all biopsies received by any pathology facility. This diverse group of lesions can exhibit a variety of presentations, ranging from a small innocuous lesion that may be discovered by chance, to a highly aggressive and destructive lesion that can even turn malignant. Among the various odontogenic cysts, the odontogenic keratocyst (OKC) in the maxillofacial region is known for its varied biological behaviors and recurrence patterns [1]. Despite advances in research, no consensus has yet been reached on the biological behaviors of this uncertain entity [2]. According to a recent study, OKCs are the third most common odontogenic cyst among Indians [3]. The diagnosis of OKC is based on histological criteria proposed by Pindborg and Hansen in 1962, with an epithelial characterization of tall columnar basal cells with focal reverse polarity [4,5].

Several studies report the chances of OKC recurrence due to the following factors: (a) the partial removal of a cystic lining; (b) the thin and friable nature of the epithelium; (c) the increased proliferation rate of the epithelium with basal layer budding; (d) infiltrative behaviors, along with bony perforations; (e) the adhesion to soft tissues in the vicinity; (f) splitting of the supra- and subepithelial lining; (g) parakeratinized variants of OKC; (h) the presence of satellite cysts/dental lamina remnants; and (i) the presence of subepithelial hyalinization [3,6,7,8]. The recurrence rates have been recorded, ranging from 0 to 100%, when patients with nevoid basal cell carcinoma syndrome are included (NBBCS) [1]. In addition to these, a few other variables were suggested for OKC recurrence, including: age, location, size, type, significant disparities in surgical procedures, and length of follow-up [7]. Several studies employing biopsy specimens have been undertaken to examine the relationship between OKC histological traits and biological potential, using immunohistochemical proliferative and anti-proliferative markers, such as p53, Ki-67, PCNA, Bcl-2, and Bax. However, no prognostic indicators, based on clinicopathological and immunohistochemical findings to predict OKC recurrence after surgical treatment, have been identified [1,3,7,9]. Despite several clinical, surgical, and other hidden factors influencing recurrence, there is a need to develop an accurate method to predict the recurrence of OKC.

With the fast growth of computer-aided techniques in recent years, the machine learning approach plays an essential role in the detection and characterization of complex clinical conditions by exploring novel prediction algorithms. Many attempts have been made to apply automated machine vision systems using mathematical formulas, image processing, and computational algorithms, to diagnose OKCs [10]. The application of deep-learning techniques has shown promising results in the diagnosis, prognostication of disease grading, and survival prediction of oral squamous cell carcinoma [11,12,13,14,15,16,17,18]. To the best of our knowledge, the prediction model for recurrence of OKCs appears to be the first of its kind. This work aimed to develop an ensemble deep-learning-based prognostic and prediction algorithm that can detect the recurrence of sporadic odontogenic keratocysts on hematoxylin and eosin stained pathological images of incisional biopsies before treatment.

**Figure 1:**
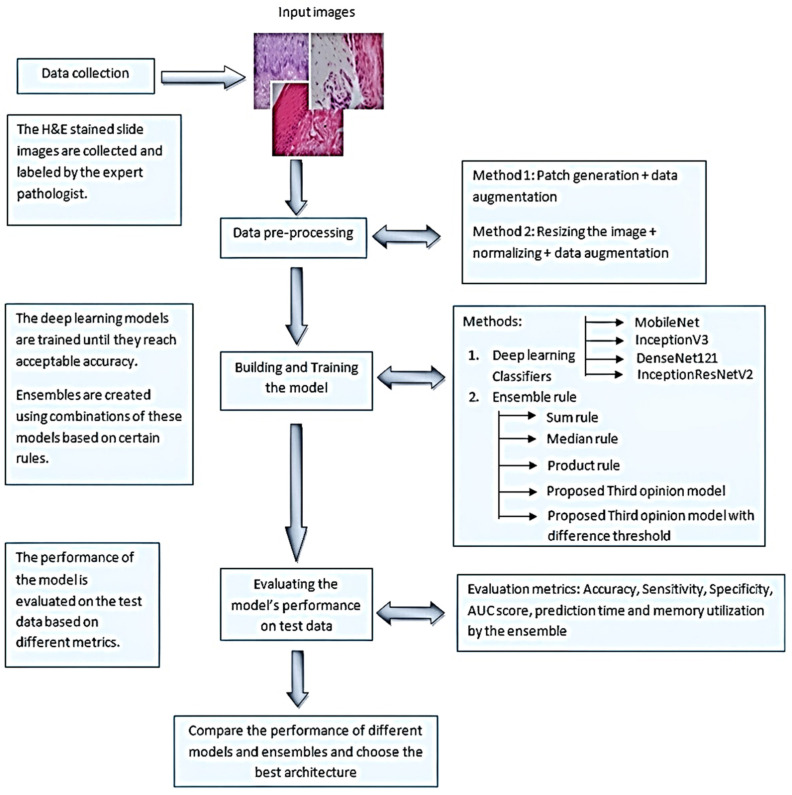
A block diagram describing the workflow of the study.

In this study, we employed deep neural networks in an ensemble with three classification models: Inception-V3, Inception-Resnet-V3, and DenseNet-121, on incisional biopsies of hematoxylin and eosin stained digitalized histopathology images to predict OKC recurrence. (Figure 1). In the ensemble model, multiple learners were combined, and a final prediction was made to determine the accuracy. We tested the efficiency of our approach over the traditional method and proved that our ensemble technique was more accurate and rapid in the detection of OKC recurrence.

## 2. Materials and Methods

### 2.1. Selection of Participants

This is a retrospective study. The protocol for this study was approved by the ethics committee of Ramaiah University of Applied Sciences (Registry Number EC-20211/F/058). Following the inclusion and exclusion criteria, all data relevant to the study objectives were gathered from a single center (Department of Oral Pathology and Microbiology, Ramaiah University of Applied Sciences, Bengaluru, India). The inclusion criteria were: (a) sporadic cases of ortho and parakeratinized variants with a minimum follow-up of five years; (b) patients with no prior treatment history, apart from being treated by the same team of surgeons with a conservative treatment protocol; and (c) a proven biopsy test of primary and recurrent OKCs of the same patient reporting in our center. The exclusion criteria included: (a) syndromic OKCs; (b) cases that underwent radical procedures; and (c) cases that lacked the required year of follow-up data. Overall, 60 participants with OKC were diagnosed and treated using a conservative manner in the facility, between the years 2009 and 2019, with a minimum 5-year post-treatment follow-up.

### 2.2. Classification of the Dataset

1660 digital slide images of OKC formalin-fixed paraffin-embedded (FFPE) tissue sectioned from 60 patient archives to five microns thickness and stained with hematoxylin and eosin were collected. The stained slides were thoroughly analyzed for histopathology features, based on the sequelae of our previous study [3]. The most reliable histological characteristics of OKCs (subepithelial hyalinization (*p* = 0.004), deficient epithelial lining (*p* = 0.023), and corrugated surface (*p* = 0.049)) as well as continuous follow-ups, were used to differentiate data status into recurrent and non-recurrent OKC (Table 1 and Table 2). The model was trained and validated on all histologically classified image data.

### 2.3. Image Characteristics

**Figure 2:**
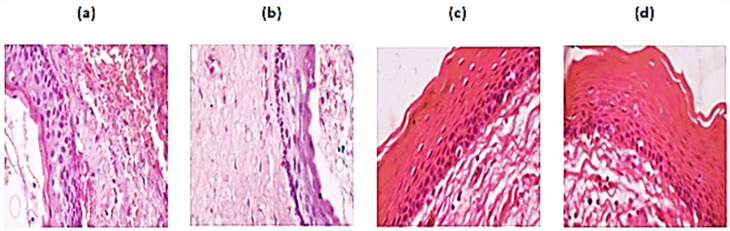
The representative samples of histopathological slides of recurrent ((**b**) subepithelial hyalinization & (**d**) corrugated surface) and non-recurrent OKC ((**a**) absence of subepithelial hyalinization & (**c**) absence of corrugated surface).

Each stained slide was magnified at 40X and captured, covering the areas of interest, using an Olympus BX53 Research Microscope (Olympus, Tokyo, Japan), with a digital Jenoptik camera and GRYPHAX imaging software (V1.1.10.6, Jena, Germany). The total dataset comprised 1660 digitalized histopathology images with 3840 × 2160 dimensions in jpg format. Among the 1660 images, 1216 belonged to non-recurring OKC and 444 to the recurring OKC group. Representative sample images of non-recurrent and recurrent OKC are shown in Figure 2. 

### 2.4. Deep Learning Classifiers and Computation

**Figure 3:**
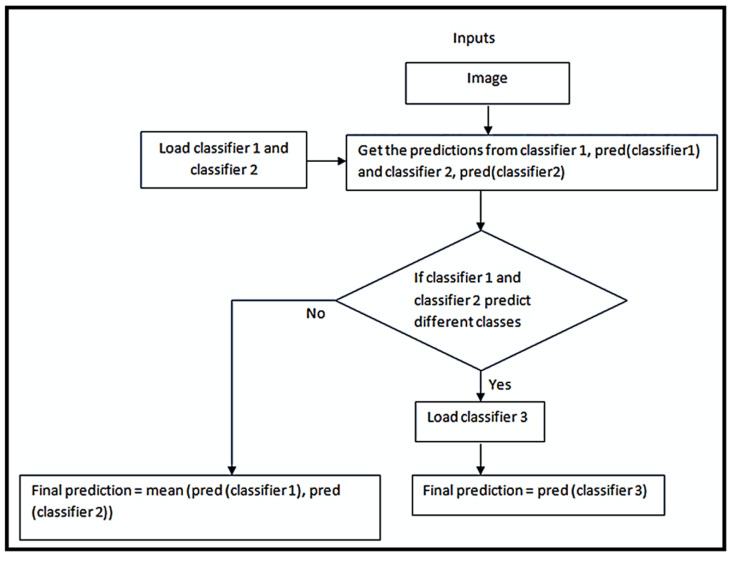
Strategy to construct a novel ensemble model.

The convolutional neural network (CNN) deep-learning model proved to be the best in image pattern classification. In addition to the requirement of a huge dataset, there were a few challenges in training the CNN: balancing an imbalanced dataset; and secondly, choosing the hyperparameters such as learning rate, batch size, network architecture, and exploding gradients. Pre-trained models could solve these issues to some extent, along with taking less time for training. We used pre-trained CNN models such as Inception-V3, DenseNet-121, and Inception-Resnet-V2, and experimented on our OKC dataset to reduce the training time. To construct the novel ensemble model, these three potential classifiers were adopted following the systematic procedure shown in Figure 3. The computations were performed using a cloud computing environment Google Colab, GPU—Tesla K80, RAM 12 GB, a personal computer (Intel(R) Core (TM) i3-4030U CPU @ 1.90 GHz), and the CNN was built with Keras.

### 2.5. Data Pre-Processing and Training

For deep-learning models, a huge dataset is typically mandatory, but was 444 and 1216 respectively. The considered dataset was imbalanced and may pose a bias on the results; to handle this, a class weight dictionary in the Keras library was utilized. This allowed the model to assign more valued weights to the class which has fewer samples than the other class. This creates more attention for the underrepresented class. The data augmentation techniques (rotation, width/height shift, shear, vertical, and horizontal flip) were implemented using the Keras ‘image data generator‘ to increase the size of the dataset. The following hyperparameters were chosen: number of dense layers, batch size, number of epochs, and learning rate (Table 3). The dataset was split into training, testing, and validation sets, where 70% of the samples were used for training, and 15% of the samples were used as a test set; the remaining samples were kept for validation. The trainable parameter for each of these derived layers was set to false before the training. The Adam optimizer was used, and the loss function was set as binary cross-entropy. The model checkpoint was used for each model training to ensure the best model had the minimum loss. Finally, the performance of each model was evaluated based on the confusion matrix, accuracy, receiver operator characteristic curve (ROC), and area under the ROC curve (AUC).

### 2.6. Deep Learning Model Classifiers

DenseNet-121 had one layer with a 7 × 7 filter mask, 58 layers with a 3 × 3 filter mask, and 61 layers with a 1 × 1 filter mask. For dimensionality reduction, four average pooling layers were used for classification. Similarly, Inception-V3 had 48 layers with 3 × 3 convolution layers. These convolution layers fetched the histopathology features, and fully connected layers were used to classify the images. Likewise, the Inception-ResNet-V2 was the combination of the Inception architecture and residual connections with 164 layers used for classification.

### 2.7. The Traditional Ensemble Models

Traditional ensemble rules were adopted by the fusion function using the sum and product rule. The sum rule takes the average of the predictions given by the classifiers (DenseNet-121, Inception-V3, and Inception-Resnet-V2) in the ensemble as an outcome of the final prediction. However, in the product rule, the product of the predictions from the classifiers was the final prediction to determine the performance. Notably, the traditional ensembles mentioned here can be computationally expensive while performing predictions on simple data. This could be experienced when these models are incorporated into a real-time application, where the prediction time increases with the number of classifiers in the ensemble.

### 2.8. Novel Ensemble Model

In this model, two of the three classifiers in the ensemble were initially loaded. At each input data point, they were checked for diverse opinions. If the predictions of the two classifiers were different, the third classifier was loaded and its decision was considered final, or else, the mean of the predictions was considered. The strategy describing this process is given in the Algorithm 1, and Figure 3 shows the flowchart of the novel ensemble model. The advantages of the novel model were that it was comparatively less computationally expensive than the traditional ensemble models, and at the same time, the ensemble effect increased the performance of the model.
**Algorithm 1** A novel ensemble modelFunction ensemble_model (X_test):        load classifier_1        load classifier_2     for each sample in X_test:        p1 = prediction from classfier_1        p2 = prediction from classifier_2          if p1 and p2 predict different classes:        load classifier_3 if not already loaded        final_prediction = prediction from classifier_3     else        final_prediction = mean of p1, p2

## 3. Results

### 3.1. Evaluating the Model’s Performance

**Figure 4:**
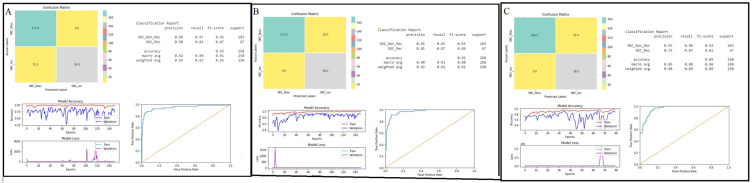
Performances of the deep-learning classifiers: (**A**) DenseNet-121, (**B**) Inception-ResNet-V2, and (**C**) Inception-V3 were demonstrated using confusion matrix, classification report for accuracy, area under ROC curve, model accuracy (accuracy vs. epochs), and loss (loss vs. epochs) plots.

The performance of the models was computed based on a confusion matrix, accuracy, and area under the ROC curve. All the classifiers well-performed showing an accuracy of more than 80%. Particularly, DenseNet-121 and Inception-V3 outperformed the Inception-ResNet-V2 models (Table 4). Both the DenseNet-121 and Inception-V3 models showed almost equal accuracies in the validation set, at 93% and 92%, respectively. Inception-ResNet-V2 achieved 90% accuracy in identifying recurrent (OKC_rec) and non-recurrent OKCs (OKC_Nrec) from images. The detailed performances of the base classifiers are shown in Figure 4A–C. In addition to the major performance characteristics, the plots in Figure 4A–C show: (1) model accuracy (accuracy vs. epochs), and (2) model loss (loss vs. epochs). Model losses were assessed, which report the behavior of the models during training and validation.

### 3.2. Ensemble Models

**Figure 5:**
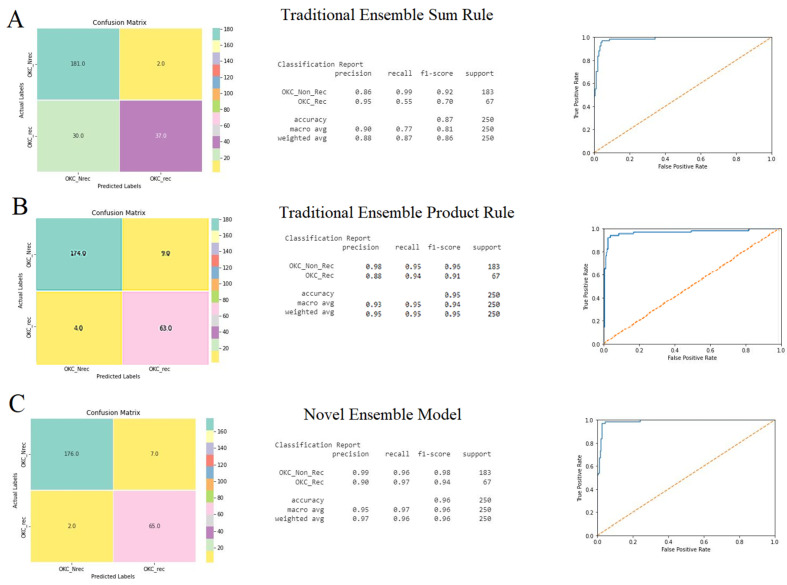
Performances of the ensemble models: (**A**) traditional ensemble sum rule, (**B**) traditional ensemble product rule, and (**C**) novel ensemble model was demonstrated using confusion matrix, classification report for accuracy, and area under ROC curve.

Considering the performances of the base classifiers, a novel ensemble model was developed and compared with the traditional ensemble models. The performance of the traditional ensemble models was computed for the sum rule and product rule, based on the confusion matrix, accuracy, and ROC curve. The ensemble model based on the sum rule showed better performance, with an accuracy of 95%, compared with the product rule with an accuracy of 88% (Figure 5A,B). Simultaneously, the novel ensemble model followed the strategy mentioned in the methodology section. Initially, Inception-V3 and Inception-Resnet-V2 were loaded, and later, DenseNet-121 was loaded. The performance had an accuracy of 97% (Figure 4C), which was more efficient than the base classifiers and traditional ensemble models, and took relatively less time (Table 4).

## 4. Discussion

Based on this investigation, we observed that our proposed deep-learning-based multi-model ensemble technique produces satisfactory results in the classification of OKC into recurrent and non-recurrent statuses. Additionally, the predictions based on the single classifiers, as well as the traditional ensemble, were efficient, producing an accuracy between 85% and 93% in the classification of the dataset. However, our novel ensemble model was outperformed by other analyzed models, including the traditional ensemble. Due to the variability in histopathology across the patients and the high recurrence of OKC, a timely and more accurate method is needed to predict the chance of OKC recurrence from the histopathological images. The possibility of predicting the invasive nature of a lesion can prevent both permanent damage to nerve structures and repeated surgical interventions [19].

With the fast development of computer-aided techniques in recent years, machine learning methods are playing an increasingly essential role in disease detection. Several researchers are constantly exploring new prediction algorithms. To the best of our knowledge, there is no artificial intelligence (AI)-based predictive model for classifying histopathological H&E-stained specimens of recurring OKC and non-recurring OKC. However, there are other AI-based predictive models that are available for oral cancer. In this study, we made a comparison between multiple model ensemble methods. We incorporated three different deep-learning classifiers, which were previously proven to be efficient. Among the three deep-learning classifiers, DenseNet-121 showed the highest accuracy at 93%, when compared with Inception-V3 and Inception-ResNet-V2. The architecture of DenseNet was inspired by a study that showed that convolutional neural networks with short connections between the layers, near the input and output layers, are efficient and accurate [18]. Additionally, within each layer of the DenseNet-121 model, the current layer′s feature maps are concatenated with those from all the previous levels [20]. Therefore, the convolutional layers contain fewer channels, the number of trainable parameters is reduced, and the model is computationally efficient. Likewise, the Inception-V3 model showed 92% accuracy, which is a relatively similar outcome to the DenseNet-121 model. Although all three models were efficient, based on their solo outcomes, we looked to improve the accuracy by intergrading these classifiers to develop an ensemble with the novel strategy, and then compared it with the traditional ensemble model.

The traditional ensemble models such as the sum, product, and median rules are popular and have the potential for use in classification problems. Herein, three deep-learning models were trained and loaded in the initial stage itself, which has a longer execution time and greater memory [17]. Finally, the prediction accuracy was obtained based on the sum and product rules, respectively. Improved accuracy of 95% was noticed using the sum rule, compared with the product rule model, and it had an execution time of ~192 s. However, adopting the novel strategy of the ensemble model showed the best performance at 97% accuracy, with an AUC of 0.98 and a significantly quicker time of 154.6 s. Compared to the traditional model, the presented novel model gave a good trade-off between accuracy and execution-time reduction, because it loads only two trained models in the initial stage. Compared with the traditional model, for the proposed models, the order of the classifiers was crucial to ensure the best accuracy and low computational cost. In this study, the classifier with the highest accuracy was chosen as the third classifier, following the given algorithm for better prediction.

Overall, the novel ensemble model took significantly less time (154.6 s) and gave an accuracy of 96% and an AUC of 0.98. This model has shown a good trade-off between the computation time and other evaluation parameters, such as memory utilization and accuracy of all the models. For future investigations, researchers may use whole slide images and validate a larger sample size to improve the accuracy and execution time.

This study provides a significant contribution to predicting the recurrence of OKC that facilitates mass screening, low-cost, and fast second opinions for critical cases; however, it is a challenging task to design and develop an histopathology image classification system that imitates the performance of human experts in H&E-stained tissues for detection of risk stratification.

Furthermore, the study is limited by the small sample size and the non-usage of whole slide images.

## 5. Conclusions

The application of machine learning methods showed significant clinical benefits in predicting OKC recurrence on a small chunk of biopsy, which not only helps doctors with detection but also supports them in following and predicting the prognosis and treatment outcomes of their patients. Considering the outcome of our novel ensemble model, based on accuracy and execution time, the presented design could be embedded into a computer-aided design system for automation of risk stratification for odontogenic keratocysts. In the future, to eliminate bias, the present study could be validated on a larger sample size of OKCs, and at different modes of treatment.

## Figures and Tables

**Table 1:** Histopathological features noted in recurrent and non-recurrent OKCs.

Histopathological Features	Recurrent OKCs		Non-Recurrent OKCs	
	Present (%)	Absent (%)	Present (%)	Absent (%)
**Subepithelial hyalinization**	75	25	35	65
**Lining (complete)**	0	100	25	75
**Lining (incomplete)**	100	0	75	25
**Keratinization (orthokeratinized)**	50	50	35	65
**Keratinization (parakeratinized)**	50	50	62.5	37.5
**Keratinization (mixed)**	0	100	2.5	97.5
**Keratin layer (thin)**	45	55	50	50
**Keratin layer (thick)**	45	55	50	50
**Keratin layer (mixed)**	10	90	0	100
**Corrugated surface**	70	30	92.5	7.5
**Folding of epithelium**	60	40	60	40
**Intracellular edema**	35	65	40	60
**Reversed polarity**	30	70	25	75
**Basilar hyperplasia**	50	50	35	65
**Rete pegs**	20	80	10	90
**Palisading**	90	10	95	5
**EPI/CT separation**	90	10	85	15
**Basal off-shoots**	30	70	17.5	82.5
**Daughter cysts**	35	65	20	80
**Inflammation (nil)**	45	55	32.5	67.5
**Inflammation (mild)**	20	80	42.5	57.5
**Inflammation (severe)**	35	65	25	75

**Table 2:** Comparison of correlation of histologic parameters with recurrent OKCs.

Histologic Parameters		Recurrence		χ^2^	*p*-Value
		Present	Absent		
**Subepithelial hyalinization**	**Present**	51.7%	48.3%	8.543	**0.004**
	**Absent**	16.1%	83.9%		
**Lining**	**Complete**	0.0%	100%	6.000	**0.023**
	**Incomplete**	40.0%	60.0%		
**Keratinization**	**Ortho**	41.7%	58.3%	1.607	0.448
	**Para**	28.6%	71.4%		
	**Mixed**	0.0%	100.0%		
**Thickness of lining**	**Thin**	31.0%	69.0%	4.138	0.126
	**Thick**	31.0%	69.0%		
	**Mixed**	100.0%	0.0%		
**Folding of epithelium**	**Present**	33.3%	66.7%	0.0	1.000
	**Absent**	33.3%	66.7%		
**Corrugated surface**	**Present**	27.5%	72.5%	5.294	**0.049**
	**Absent**	66.7%	33.3%		
**Intercellular edema**	**Present**	30.4%	69.6%	0.141	0.783
	**Absent**	35.1%	64.9%		
**Reversed polarity**	**Present**	37.5%	62.5%	0.170	0.760
	**Absent**	31.8%	68.2%		
**Basilar hyperplasia**	**Present**	41.7%	58.3%	1.250	0.280
	**Absent**	27.8%	72.2%		
**Rete pegs**	**Present**	50.0%	50.0%	1.154	0.422
	**Absent**	30.8%	69.2%		
**Palisading**	**Present**	32.1%	67.9%	0.536	0.595
	**Absent**	50.0%	50.0%		
**EPI/CT separation**	**Present**	34.6%	65.4%	0.288	0.707
	**Absent**	25.0%	75.0%		
**Basal offshoots**	**Present**	46.2%	53.8%	1.227	0.326
	**Absent**	29.8%	70.2%		
**Daughter cysts**	**Present**	46.7%	53.3%	1.600	0.223
	**Absent**	28.9%	71.1%		
**Inflammation**	**Absent**	40.9%	59.1%	2.967	0.227
	**Mild**	19.0%	81.0%		
	**Severe**	41.2%	58.8%		

Chi-squared test, *p*-value < 0.05 is statistically significant.

**Table 3:** Hyperparameters used in the models.

Hyperparameter	Classifier 1	Classifier 2	Classifier 3
Number of dense layers	3	1	4
Batch size	72	64	84
Number of epochs	82	35	57
Learning rate	0.001	0.001	0.001

**Table 4:** Comparative performance of the models.

Parameter	DenseNet-121	Inception-Resnet-V2	Inception-V3
**Performance of the base classifier**			
Accuracy (%)	93	88	92
AUC	0.9452	0.9602	0.9653
**Performance of Ensemble Models**			
	Traditional ensemble model (Sum rule)	Traditional ensemble model (Product rule)	**Novel ensemble model**
Accuracy (%)	95	88	96
Average computational time (in seconds)	192.9	198.5	154.6

## Data Availability

Not applicable.
