# Correction: Rao et al. Ensemble Deep-Learning-Based Prognostic and Prediction for Recurrence of Sporadic Odontogenic Keratocysts on Hematoxylin and Eosin Stained Pathological Images of Incisional Biopsies. *J. Pers. Med.* 2022, *12*, 1220

**DOI:** 10.3390/jpm16060334

**Published:** 2026-06-22

**Authors:** Roopa S. Rao, Divya Biligere Shivanna, Surendra Lakshminarayana, Kirti Shankar Mahadevpur, Yaser Ali Alhazmi, Mohammed Mousa H. Bakri, Hazar S. Alharbi, Khalid J. Alzahrani, Khalaf F. Alsharif, Hamsa Jameel Banjer, Mrim M. Alnfiai, Rodolfo Reda, Shankargouda Patil, Luca Testarelli

**Affiliations:** 1 Department of Oral Pathology and Microbiology, Faculty of Dental Sciences, Ramaiah University of Applied Sciences, Bengaluru 560054, India; 2 Department of Computer Science and Engineering, Faculty of Engineering and Technology, Ramaiah University of Applied Sciences, Bengaluru 560054, India; 3 Division of Oral and Maxillofacial Surgery, Department of Oral and Maxillofacial Surgery and Diagnostic Sciences, College of Dentistry, Jazan University, Jazan 45412, Saudi Arabia; 4 Department of Basic Dental Science, College of Dentistry, Princess Nourah Bint Abdulrahman University, Riyadh 11564, Saudi Arabia; 5 Department of Clinical Laboratories Sciences, College of Applied Medical Sciences, Taif University, 11099, Taif 21944, Saudi Arabia; 6 Department of Information Technology, College of Computers and Information Technology, Taif University, 11099, Taif 21944, Saudi Arabia; 7 Department of Oral and Maxillo Facial Sciences, Sapienza University of Rome, 00161 Rome, Italy; 8 Department of Maxillofacial Surgery and Diagnostic Sciences, Division of Oral Pathology, College of Dentistry, Jazan University, Jazan 45412, Saudi Arabia; 9 Centre of Molecular Medicine and Diagnostics (COMManD), Saveetha Dental College & Hospitals, Saveetha Institute of Medical and Technical Sciences, Saveetha University, Chennai 600077, India

The Author Contributions is incomplete in the original publication [1]. The corrected Author Contributions statement appears here.

**Author Contributions**

Conceptualization, R.S.R., D.B.S., R.R. and S.L.; methodology, R.S.R., K.S.M., S.P. and K.J.A.; software, H.S.A. and Y.A.A.; validation, H.J.B., M.M.A., R.R. and L.T.; formal analysis, K.J.A. and K.F.A.; investigation, K.S.M., M.M.H.B. and S.P.; resources, K.J.A. and K.F.A.; data curation, S.L. and K.S.M.; writing—original draft preparation, R.S.R., D.B.S., S.L., M.M.H.B., K.S.M., R.R., S.P. and L.T.; writing—review and editing, K.J.A., K.F.A., H.J.B., M.M.A., H.S.A., Y.A.A., R.R. and L.T.; visualization, R.S.R. and D.B.S.; supervision, R.S.R., S.L., K.S.M. and L.T.; project administration, S.P., H.J.B., M.M.H.B. and K.J.A.; funding acquisition, M.M.A., H.S.A. and Y.A.A. All authors have read and agreed to the published version of the manuscript.

**Error in Email**

In the original publication [1], there was a mistake in email for the author Hamsa Jameel Banjer as published. The corrected email changed to h.banjer@tu.edu.sa.

The authors state that the scientific conclusions are unaffected. This correction was approved by the Academic Editor. The original publication has also been updated.
